# Revised You’re Welcome Criteria and Future Developments in Adolescent Healthcare

**DOI:** 10.4274/jcrpe.v3i2.10

**Published:** 2011-06-08

**Authors:** Dougal S. Hargreaves

**Affiliations:** 1 Institute Child Health, General & Adolescent Paediatrics, London, United Kingdom

**Keywords:** General paediatrics, adolescent health, health services research Conflict of interest: None declared

## Abstract

In 2011, the Department of Health (England) will publish revised You’re Welcome criteria. This is the first comprehensive attempt to define good quality health services for young people (11-19 years) and provide a self-assessment tool applicable to all adolescent health services. It builds on a growing understanding of the distinctiveness and importance of adolescent health, and the demands placed on adolescent health services. This article reviews changing understandings of the nature of adolescence, including physical, psychological and social transition, evolving patterns of morbidity and mortality, adolescence as part of a life-course approach to health and health behaviours, and the specific needs of young people when using health services. We describe key features of the You're Welcome criteria and discuss the views of young people and professionals involved in revising them, as well as relevant published literature. Lastly, we discuss how the perspective of social paediatrics may be useful in guiding professionals towards a more holistic approach to adolescent care in the future.

**Conflict of interest:**None declared.

## INTRODUCTION

Despite the current United Nations International Year of Youth (1)  and a UNICEF focus on adolescence (2), attitudes to young people vary widely between and within societies. In Britain, negative perceptions of young people (3), including a tendency to overestimate the criminal threat presented by young people (4), are combined with widespread concerns about a ‘lost generation’, who are entering a society with fewer educational and economic opportunities than their predecessors (5,6).  Within healthcare, the last decade has seen increasing concern about the barriers that young people face in accessing healthcare and the quality of care provided (7,8). This year, the English Department of Health is publishing new standards which will allow all health services that see adolescents to assess their service against detailed quality criteria. The You’re Welcome criteria build on previous standards for primary care and community services (9) and were developed in partnership with a wide range of young people and healthcare professionals.

In this article, we review changing understandings of the nature of adolescence, including physical, psychological and social transitions, evolving patterns of morbidity and mortality, adolescence as part of a life-course approach to health and health behaviours, and the specific needs of young people when using health services. We then describe key features of the You’re Welcome standards and discuss how these relate to both published literature and the views of young people and professionals. Lastly, we discuss how the perspective of social paediatrics may be useful in guiding health professionals to a more holistic approach to adolescent care.

## DEFINITION

Adolescence is defined by World Health Organisation (WHO) as 10-19 years (10),  and the You’re Welcome criteria are intended primarily for services seeing young people from 11-19. However, many health issues are common to older teenagers and young adults. Much of the literature uses the age range 10-24 (11)  and this is supported by recent evidence that the health of young adults may be worse than that of adolescents (12).

Adolescence as a Unique Life Stage Understanding of the nature of adolescence continues to evolve. Studies have long demonstrated the impact of neuro-endocrine changes and sexual maturation on psychosocial development (13,14), but recent findings emphasise that the brain continues to mature for a decade beyond puberty (15,16), with continued development of the pre-frontal cortex and expansion of cortical-cortical communication. 

From a psychological perspective, adolescence is a time when the concept of the self, the ability to understand other’s perspectives, attitudes to risk, and susceptibility to peer influence all undergo major changes (17). Consistent with brain imaging studies mentioned above, recent findings show that the interaction between risky behaviour and the presence of peers continues to develop throughout adolescence and beyond (18).Meanwhile, the social transition to adulthood is changing too, both in the criteria that define adulthood and in increasing ambivalence about their own status by young adults (19). Data suggest that a majority of young Americans do not feel they are fully adult before their late 20s (20). 

**Epidemiology and a Life-Course Approach**

Epidemiological data suggest that views of adolescence as a healthy time of life may be outdated. With the exception of very low income countries, mortality in 15-24s is higher than any other period of childhood outside infancy. Mortality has improved at half the rate of younger groups, such that mortality in men aged 15-24 is now 2-3 times higher than in boys aged 1-4 (**11**).

The importance of adolescence for adult health is now better appreciated, with up to 75% of adult mental illness presenting before the age of 18 (21)  and globally, 45% of newly-acquired HIV infection occurring in 15-24s (**22**). Similarly, behaviours established in adolescence are linked to life-long risk in smoking (23), obesity (23), alcohol intake (24) and hyperlipidaemia (25).

While the importance of early influences are not in doubt, Marmot (26), UNICEF (2)  and others have emphasised the importance of a life-course approach to health and life opportunities, with investment in early years followed by ongoing investment throughout childhood and adolescence. A comprehensive US review (27) found that investment in early years was the most cost-effective, but that ‘remediation in the adolescent years can repair the damage of adverse early environment’. Similarly, the contribution of economic inequality to health is well-established (26,28), but an understanding of adolescence as a ‘key period in the emergence of health inequalities’ (29)  is more recent, and the picture may be complicated by the existence of other social hierarchies in this age group (30). 

Adolescent Friendly Services and You’re Welcome Alongside many other transitions, young people are expected to take responsibility for their own health, start accessing healthcare independently and, in the case of young people with a long-term condition, negotiate the transition from paediatric to adult services. The barriers that young people often face in accessing healthcare include physical and financial issues, embarrassment or lack of knowledge, concerns about stigma, confidentiality, and consent, and deterrence by an inappropriate or unfriendly service.

In 2002, WHO identified health services for young people as a priority area for improvement (7), a call mirrored by the UK Medical Royal Colleges in 2003 (31).  A Lancet review in 2007 (8) reported mixed progress overall, and identified three main approaches which had been used to improve the  performance of adolescent services: 

- provision of guidelines

- provider training

- quality-improvement strategies incorporating provide training.

Building on this literature and influenced by the UN Convention on the Rights of the Child (32), some recent policy work has placed greater emphasis on involving young people themselves in improving youth services (33,34). In 2007, the English Department of Health published the You’re Welcome quality criteria for community and primary care health services (9)  and, in 2009, an accompanying self-assessment tool (35). Services seeing young people were encouraged to assess their services against these criteria and then work with young people who used the service to ask their views and improve areas of weakness. They could then apply to be certified as meeting the criteria. 

The criteria proved popular, with support from professional bodies (36), youth groups and the National Health Service Operating Framework (37). By March 2011, over 100 services had been formally accredited, with many more engaging but still in the process of improving their services. Commissioners were also supportive, with some providing additional funds to services in order to work towards You’re Welcome accreditation. 

In 2009, work was started to revise the criteria so that they would be applicable to all health services seeing young people, including acute and specialist services. 16 sites were recruited to the project, with a mixture of specialist children’s hospitals, large teaching hospitals, smaller district general hospitals and two hospices. The criteria were then reviewed by staff and young people at each site, with discussion and sharing of findings at 3 national workshops.

The revised criteria will be available via the Department of Health website (38).  Rather than duplicate the criteria, the following section is intended as a discussion of relevant published literature and the views of staff and young people who took part in the consultation process. The main 8 criteria are discussed while, for reasons of space, issues specific to sexual and mental health are not included. Where not otherwise referenced, examples and views are from professionals and young people at the 16 project sites.  To encourage participation, permission was not sought to publish the names of participants or details of specific services and most examples are therefore anonymised. The consultation process was intended to guide policy development; further consultation using rigorous research methods and leading to publication would be welcome.

**You’re Welcome Criteria**

**1. Access**

Being able to access healthcare without excessive practical, financial, or self-imposed barriers is fundamental to all further discussion on the quality of care provided. While the literature in the US often focuses on insurance coverage and financial barriers (12), much of the wider international debate relates to wider barriers, including delay in seeking care due to embarrassment or anxiety about confidentiality or judgemental attitudes by staff (8). Access is related to patient satisfaction, with young people who report satisfaction with a service saying they are more likely to attend for follow-up (39). In the UK, one study found no major differences in young people’s use of healthcare by socio-economic status (after adjusting  for perceived health status) (40)  but reported more frequent  general practitioner (GP) consultations among South Asians and more use of hospital services by White young people. However, in-depth work with the most vulnerable young people has reported that there can be significant  difficulty in accessing appropriate services for specific groups 41).Optimising access depends on the local context. In our consultation, young people in a rural area reported being more dependent on their parents for transport and were more likely to value sexual or mental health services on school premises, which they could access without their parents’ knowledge. Conversely, some young people in an urban environment reported frequent use of public transport and found more privacy in services provided outside school. Specialist services may have to be more creative; telecare consultations using a patient’s home computer may be used increasingly in the future. 

Much discussion in the development of the You’re Welcome criteria concerned the tension between ideal or best practice and financial or practical constraints. For some issues (e.g. counselling in early pregnancy), there was wide consensus that the ethical views of individual professionals’ should not limit the choices of their patients, and alternative arrangements should be made where necessary.

There was more discussion around seeing young people alone, for at least part of the consultation. This is widely considered good practice (9,42), and supported by young people in our consultations, although previous consultations have shown some ambivalence by young people (43). However, it is a change in practice for many general practitioners and paediatricians and some areas reported colleagues who felt uncomfortable providing this service. In a hospice setting, some staff felt that this was either not always appropriate in end of life care, or simply not practical to provide (for example when the mother of a young man provided all of his transport and declined to bring him to a group workshop on sexual health). Lastly, expressing a preference for the gender of the professional seen and being accompanied by a friend were seen as more negotiable. They were highly valued in certain contexts (e.g. attending a sexual health clinic for the first time) but sometimes impractical in others (e.g. attending a specialist clinic, led by one consultant).

**2. Publicity**

Although sometimes seen as a peripheral issue by medical staff, young people consistently mentioned the importance of publicity material in influencing their decisions of where to access healthcare and what to expect when they did. Creating this material with young people was sometimes a good way of engaging young people and could have a wider impact on young people’s awareness of health issues locally (44).

**3. Confidentiality/Consent**

Confidentiality is a frequent source of anxiety in adolescent healthcare – both for young people and professionals. UK guidance states that young people under 16 can and should be treated in confidence if this is in their best interests and they are deemed competent to make their own decisions (45,46). However, confidentiality should not always be respected if doing so would put the young person or others at risk of harm or if there are over-riding legal or public interest reasons not to do so. A related area is the autonomy of adolescents in giving or withholding consent to treatment. A recent editorial by Duncan and Sawyer (47) discussed that doctors were increasingly likely to respect the autonomy of young people but only to the extent that they make what is perceived to be the ‘right choice’. UK courts have sometimes supported the clinical judgement of doctors over the wishes of young patients (48)  and current General Medical Council guidance is for doctors to seek legal advice where there is dispute (45). This raises interesting psychological and philosophical questions about individual responsibility and autonomy, particularly in the light of research mentioned above regarding brain development (15)  and in comparison with the age of criminal responsibility (49). Consistent with previous findings (41), young people in our project sites valued  professionals taking the time to explain confidentiality. An honest discussion of the limits of confidentiality showed respect and helped to build their trust in the service. As mentioned above, clear communication about confidentiality in publicity material allayed anxiety and made them more likely to attend. Many professionals also felt that communication around confidentiality could be improved in their service and welcomed further training and peer support in this area.  

**4. Environment**

First impressions are important: a waiting room with magazines and posters of interest to teenagers can create an impression of a service for ‘people like me’, while a selection of toys for young children or a room full of elderly and unwell people can be very off-putting. Improving the waiting room can also be a relatively easy way to engage young people and raise the profile of adolescent health within a hospital or other setting.

However, You’re Welcome interprets the environment more widely to include the atmosphere and culture of delivering age-appropriate care. For example, warmth, privacy and confidentiality need to be maintained throughout the patient journey; some young people complained that, regardless of professionals’ actions, a receptionist who asked questions in front of others or was seen as unfriendly, deterred them from attending the next time.Many young people felt that the needs of adolescents are so distinct from those of younger children that they should be provided in dedicated adolescent units. In the UK, this is  unusual outside large teaching hospitals, despite evidence that they are rated highly by young people and their parents (50). The compromise of an adolescent section within the paediatric ward (preferred by most young people, regardless of the sex of other patients) was sometimes in conflict with government targets to stop mixed-sex bays for all ages.

**5. Staff Training, Skills and Values**

Negative stereotypes about teenagers are widespread in the UK (3) and healthcare staff are not exempt from this. One young person said the most important healthcare issue for him and his friends was being seen by ‘people who like us’. However, it is often poor communication, rather than simple dislike, which prevents better consultations for young people. A randomised controlled trial among Australian general practitioners showed that a short course in communicating with adolescents increased confidence of doctors and satisfaction of young people significantly (51). Despite potential embarrassment, young people value the opportunity to discuss sensitive topics such as sexual health, substance misuse, and mental health issues and rate the quality of the consultation more highly if these are discussed (52). Standardised tools such as HEADSS (53) provide a framework for trainees and non-specialists to gain confidence in discussion sensitive topics. Wider training initiatives include e-learning programmes in adolescent health (EU teach (54), and the Adolescent Health Programme (55) - accessible free to UK health professionals) and a new curriculum for trainees in general paediatrics who wish to develop an interest in adolescent health (56). 

**6. Joined-up Working**

With the increasing complexity of health systems, a common complaint among young and old patients is the lack of care co-ordination and poor communication between different professionals. Navigating the bureaucracy of referral systems is a major deterrent to accessing care for young people and the recent Kennedy review identified this as a particular failing of  National Health services for young people with complex needs (57). Many services, especially for the most vulnerable young people, aim to provide a ‘one-stop shop’ where a full range of basic health service are co-located with other support services for young people (41). Possible improvements in communication range from the simple (sending copies of clinic letters to young people and/or their parents) to more sophisticated systems to share information. For example, the company Patients Know Best (58) uses a social-network-style platform whereby a patient can invite a range of professionals to join his/her personal network, thereby giving consent for them to share information with each other. In many organisations, better information sharing will only come for young people as part of a broader improvement for all patients. However, young people’s confidence with technology means they may be at the forefront of these advances. 

**7.  Young People’s Involvement in Monitoring and Evaluation of Patient Experience** 

This lies at the heart of the You’re Welcome approach. It builds on the government ambitions for a more patient-centred NHS for all ages (59,60), is consistent with professional guidance (61,62), legal obligations under Article 12 of the United Nations Convention on the Rights of the Child (UNCRC) (32), and the views of young people themselves (41,63,64). Hart (65) identifies potential dangers in participation projects and these are discussed in the  context of health services in a recent publication by the Royal College of Paediatrics and Child Health (61).

Some project sites benefited from the growing trend towards hospital-based youth workers (66), who have particular expertise and experience in promoting youth participation. However, nurses and doctors successfully led participation projects in other sites. Alongside traditional means of involving young people such as surveys and youth groups, public panel discussions and social networking sites were also used (67). Other techniques needed to be matched to the local context. For example, mystery shopping has been successfully used to assess the quality of consultations in GU clinics (68). In a general hospital setting, mystery shopping was found to be a good way of assessing some aspects of care, including the friendliness of receptionists, the atmosphere of the waiting room, and ease of wheelchair access. However, none of the project sites used it to assess the quality of clinical consultations with young people. Concerns were sometimes raised around issues of representation, with prospective medical students and those with long-term conditions over-represented while users of sensitive services (for example sexual health and abortion services) and ‘well’ young people under-represented. However, one positive finding was that many groups included young people from a wide range of ethnic and socio-economic backgrounds, especially relative to adult patient representatives.

**8. Health Issues and Transition of Young People**

This section puts the individual needs of the young people at the centre and considers a range of issues which contribute to their overall experience of health services. First is the opportunistic use of consultations for health promotion and provision of other services (e.g. sexual health, smoking cessation, immunisation, identification of emotional and mental health needs). In some cases, this may require extra training or support in order to provide these services in-house and avoid referrals for minor issues. An emphasis on health promotion reflects the epidemiology and life-course perspective of adolescence – where risky behaviour and mental health issues have a great impact on current and future health, while physical illness may be less important than in the old or very young (11). In reality, the transition to young adulthood is often marked by increasing use of Emergency Departments to access care and, at least in males, infrequent use of preventative services (69). The American Academy of Paediatrics recommends routine annual check-ups for adolescents (42), while English policy recommends at least one review in the mid-teens, combined with other efforts to engage young people, such as a ‘birthday card’ on their 16th birthday, informing them of the services available and inviting them to make an appointment (70).  A previous consultation found that some young people would prefer to see a school nurse and others their GP, largely depending on where they had had positive experiences previously (43). For those with a long-term condition or other specialist needs, the process of transition to adult services is often a source of anxiety and may end with disillusionment and disengagement with health services (57). In many cases, the process is complicated by the social transition, as the young person moves out of the family home and may move geographically for work or study. Improving this process is important, not only to avoid psychological distress, but to secure attendance at adult clinics and improve medical outcomes. The period of transition has been linked to concerns in a range of conditions, including failure of renal transplant, and poorer control of diabetes, cystic fibrosis, and juvenile arthritis (71). Best practice considers transition as a process, not an event, involving preparation and a degree of continuity over many years. A holistic approach should consider the condition in the context of the person’s family, school, work and other commitments, and should take account of individual needs and preferences (72). As with all areas of adolescent health, the chronological age should be considered alongside their cognitive ability and level of emotional maturity. The importance of social influence in adolescence is reflected in the enthusiasm for peer support and education. The Young Expert Patient Programme (73)  and Getting Sorted (74)   are two popular programmes that support young people with long-term conditions in gaining the confidence and skills to take responsibility for their own health and make a successful transition to adult services. 

**Adolescent Health and Social Paediatrics**

The You’re Welcome criteria aim to show that it is both important and realistic to improve the quality of healthcare for adolescents. They rest on new understandings of the nature of adolescence, current epidemiology, the importance of adolescence in determining lifetime risk behaviours, and reflect modern healthcare delivery. Although developed in the context of England, principles of adolescent-friendly care are important internationally, for example being identified by WHO Africa as an important part of the response to HIV/AIDS (75)  and the Pan American Health Organisation as important in tackling social inequalities in health (76). However, improving health services is clearly only part of the process of improving adolescent health. McGinnis et al (77) found that healthcare has an important but limited ability to improve population health, and this  message has been reinforced by the evidence around  economic inequality and wider social determinants of health (26). A wider approach is that of Social Paediatrics, defined as an approach to child health that focuses on the child, ‘in illness and in health, within the context of their society, environment, school, and family’. Although in the UK, it has sometimes been interpreted as ‘protection of children from abuse and children who are adopted or fostered’ (78), the European Society for Social Paediatrics (ESSOP) defines social paediatrics much more broadly, including advocacy for social justice, education and training, and provision of health promotion, preventative and curative healthcare (79). Social Paediatrics offers a framework within which paediatricians have succeeded in advocating on issues of economic injustice (80), used advances in neurosciences to raise awareness and improve services (81), and developed training programmes which integrate acute clinical care and wider action to promote child health and well-being in their local communities (82). Although the details may be different in working with young children and adolescents, the philosophy matches the calls by young people themselves for an approach that ‘sees me, not just my illness’. Adolescent health professionals, working in partnership with the young people they serve, are well aware of the scale of challenges facing them. We propose that the combination of specific tools such as the You’re Welcome criteria and holistic approaches such as Social Paediatrics may offer potential for significant improvements in the future. 

**Acknowledgements**

I thank all the young people and professionals at the 16 project sites, as well as colleagues from the Young People’s Health Special Interest Group, the Association of Hospital-Based Youth Workers, and the Department of Health, who all contributed to the consultation process and the revised You’re Welcome criteria. Competing Interests Dougal Hargreaves was employed by the Department of Health (England) as Clinical Advisor from March 2009 – March 2011, and contributed to the revised You’re Welcome criteria. However, the views expressed are entirely his own and do not represent Department of Health policy. 

I declare no conflict of interest.

## References

[1] (2011). United Nations International Year of Youth August.

[2] (2011). UNICEF State of the World's Children 2011 – Adolescence: An Age of Opportunity..

[3] (2009). Scottish Government. UN Convention on the Rights of the Child: Scottish Governments detailed response to the UN Committees 2008 Concluding Observations..

[4] (2008). Halsey K, White R. Young People, Crime and Public Perceptions: a Review of the Literature(LGA Research Report F/SR264).  2008. Slough, NFER..

[5] (2009). Blanchflower D. This lays bare the human crisis. What a terrible time to be young and jobless..

[6] (2010). National Union of Students. A-Level Results - Door of opportunity slammed in the face of the lost generation.

[7] (2002.). Adolescent friendly health services: an agenda for change.  2002..

[8] Tylee A, Haller DM, Graham T, Churchill R, Sanci LA. (2007). Youth-friendly primary-care services: how are we doing and what more needs to be done?. Lancet.

[9] (2011). Department of Health (England). You're Welcome quality
criteria: Making health services young people friendly..

[10] (2011). WHO.

[11] Viner RM, Coffey C, Mathers C, Bloem P, Costello A, Santelli J, Patton GC (2011). 50-year mortality trends in children and young people: a study of 50 low-income, middle-income, and high-income countries.. Lancet.

[12] Irwin CE Jr. (2010). Young adults are worse off than adolescents.. JJ Adolesc Health.

[13] Rutter M, Graham P, Chadwick OF, Yule W. (1976). Adolescent turmoil: fact or fiction?. J Child Psychol Psychiatry.

[14] Jones MC. (1965). Psychological correlates of somatic development.. Child Dev.

[15] Patton GC, Viner R (2007). Pubertal transitions in health. Lancet.

[16] Sowell ER, Thompson PM, Holmes CJ, Jernigan TL, Toga AW (1999). In vivo evidence for post-adolescent brain maturation in frontal and striatal regions. Nat Neurosci.

[17] Sebastian C, Burnett S, Blakemore SJ. (2008). Development of the self-concept during adolescence.. Trends Cogn Sci.

[18] Gardner M, Steinberg L (2005). Peer influence on risk taking, risk preference, and risky decision making in adolescence and adulthood: an experimental study.. Dev Psychol.

[19] Nelson LJ BC. (2005). Distinguishing Features of Emerging Adulthood The Role of Self-Classification as an Adult.. Journal of Adolescent Research March.

[20] Arnett JJ. (2000). Emerging Adulthood. A  Theory  o f  Development From  the  Late  Teens  Through  the  Twenties.. Am Psychol.

[21] (2011). Department of Health (England). No health without mental health: a cross-Government mental health outcomes 
strategy for people of all ages.

[22] (2011). WHO.

[23] (2008). Office for National Statistics (Great Britain). General Lifestyle Survey, 2008.

[24] (2008). Donaldson L. Under their skins:Tackling the health of the teenage nation. Chief Medical Officer's Annual Report 2007..

[25] Magnussen CG, Thomson R, Cleland VJ, Ukoumunne OC, Dwyer T, Venn A. (2011). Factors affecting the stability of blood lipid and lipoprotein levels from youth to adulthood: evidence from the Childhood Determinants of Adult Health Study.. Arch Pediatr Adolesc Med.

[26] (2010). Marmot M. Fair Society, Healthy Lives: Strategic Review of Health Inequalities in England post 2010..

[27] Heckman JJ. (2008). Schools, skills, and synapses. Econ Inq. Econ Inq.

[28] (2009). Wilkinson R, Pickett K. The Spirit Level: Why Equality is Better for Everyone.

[29] (2008). Currie C. Inequalities in young people's health: HBSC International Report from the 2005/2006 Survey.  ..

[30] West P, Sweeting H, Young R, Kelly S. (2010). The relative importance of family socioeconomic status and 
school-based peer hierarchies for morning cortisol in youth: an exploratory study.. Soc Sci Med.

[31] (2003). The Intercollegiate Working Party on Adolescent Health. Bridging the Gaps: Health Care for Adolescents.

[32] (1990). Article 12, United Nations Convention on the Rights of the Child.

[33] (2011). New South Wales Centre for Advancement of Adolescent Health (NSW CAAH). ACCESS Study: Youth Health – Better Practice Framework Fact Sheets.

[34] (2009). Europe. Meeting on youth-friendly health policies and services, Edinburgh, Scotland. 2009..

[35] (2009). Department of Health (England). You're Welcome quality criteria self-assessment toolkit.

[36] (2011). Young People's Health Special Interest Group (part of the Royal College of Paediatrics and Child Health)..

[37] (2008). Department of Health (England). The operating framework for the NHS in England 2009/10..

[38] (2007). Department of Health (England). You're Welcome quality criteria: Making health services young people friendly. London.

[39] Freed LH, Ellen JM, Irwin CE Jr, Millstein SG. (1998). Determinants of adolescents' satisfaction with health care providers and intentions to keep follow-up appointments.. J Adolesc Health.

[40] Cooper H, Smaje C, Arber S. (1998). Use of health services by children and young people according to ethnicity and social class: secondary analysis of a national survey.. BMJ.

[41] (2009). Sawtell M. Innovative services to improve the health and wellbeing of vulnerable young people: Evaluation examples from Teenage Health Demonstration Sites..

[42] Achieving Quality Health Services for Adolescents Committee on Adolescence
 (2008). Committee on Adolescence of American Academy of Paediatrics. Achieving quality health services for 
adolescents.. Pediatrics.

[43] (2011). Think Knowsley.

[44] (2010). General Medical Council. 0-18 years: Guidance for all 
doctors.

[45] (2011). British Medical Association. Children and Young People Toolkit.

[46] Duncan RE, Sawyer SM. (2010). Respecting adolescents' autonomy (as long as they make the right choice).. J Adolesc Health.

[47] Bridge C (1999). Religious beliefs and teenage refusal of medical treatment.. Mod Law Rev.

[48] Steinberg L, Scott ES. (2003). Less guilty by reason of adolescence: developmental immaturity, diminished responsibility, and the juvenile death penalty.. Am Psychol.

[49] Viner RM. (2007). Do adolescent inpatient wards make a difference? Findings from a national young patient survey.. Pediatrics.

[50] Sanci LA, Coffey CM, Veit FC, Carr-Gregg M, Patton GC, Day N, Bowes G. (2000). Evaluation of the effectiveness of an educational intervention for general practitioners in adolescent health care: randomised controlled trial..

[51] Brown JD, Wissow LS. (2009). Discussion of sensitive health topics with youth during primary care visits: relationship to youth perceptions of care.. J Adolesc Health.

[52] Cohen E, Mackenzie RG, Yates GL. (1991). HEADSS, a psychosocial risk assessment instrument: implications for designing effective intervention programs for runaway youth.. J Adolesc Health.

[53] (2009). European Training in Effective Adolescent Care and Health (EuTEACH) Programme..

[54] (2011). Adolescent Health Programme..

[55] (2010). Royal College of Paediatrics and Child Health. A Framework of Competencies for the Level 3 Training  Special Study Module in  Adolescent Health..

[56] (2010). Kennedy I. Getting it right for children and young people: Overcoming cultural barriers in the NHS so as to meet their needs.

[57] (2011). Patients Know Best.

[58] (2010). Department of Health (England). Equity and excellent: Liberating the NHS..

[59] (2010). Department of Health (England).  Achieving Equity and Excellence for Children..

[60] (2010). Wood DTGSF. Not Just a Phase: A Guide to the Participation of Children and Young People in Health Services..

[61] (2010). OFSTED. Supporting young people: An evaluation of recent reforms to youth support services in 11 local areas.

[62] (2011). Adams L ASBJBJFSFSeal. 'Nothing about us, without us?' Young people and the future of the NHS'.

[63] (2011). National Youth Agency – Hear by Right..

[64] (2011). Hart R. Children's Participation from Tokenism to Citizenship.  1992. Florence, UNICEF Innocenti Research Centre. 66. Hospital youth workers 4 health..

[65] (2011). Hospital youth workers 4 health.

[66] (2011). You Get Me Facebook page..

[67] Weston R, Dabis R, Ross JD. (2009). Measuring patient satisfaction in sexually transmitted infection clinics: a systematic review.. Sex Transm Infect.

[68] Callahan ST, Cooper WO. (2010). Changes in ambulatory health care use during the transition to young adulthood.. J Adolesc Health.

[69] (2009.). Department of Health (England). Healthy Child Programme From 5-19 years old..

[70] (2011). O'Donoghue D. Smoothing the transition to adult health care. Eyes on Evidence [22]..

[71] (2011). WHO Africa..

[72] Viner R. (2001). Barriers and good practice in transition from 
paediatric to adult care.. J R Soc Med.

[73] (2011). Young Expert Patient Programme..

[74] (2008). Pan American Health Organization. Regional Strategy for Improving Adolescent and Youth Health..

[75] Spencer N; ESSOP. (2008). European Society for Social Pediatrics and Child Health (ESSOP)* Position Statement: Social inequalities in child health - towards equity and social justice in child health outcomes.. Child Care Health Dev.

[76] McGinnis JM, Williams-Russo P, Knickman JR. (2002). The case for more active policy attention to health promotion.. Health Aff (Millwood).

[77] Ford-Jones EL, Williams R, Bertrand J. (2008). Social paediatrics and early child development: Part 1.. Paediatr Child Health.

[78] Spencer N, Colomer C, Alperstein G, Bouvier P, Colomer J, Duperrex O, Gokcay G, Julien G, Kohler L, Lindström B, Macfarlane A, Mercer R, Panagiotopoulos T, Schulpen T. (2005). Social paediatrics.. J Epidemiol Community Health.

[79] (2011.). European Society for Social Paediatrics.

[80] (2009). Scottish Government. UN Convention on the Rights of the Child: Scottish Governments detailed response to the UN Committees 2008 Concluding Observations..

[81] (2011). DH.

[82] Guyda H, Razack S, Steinmetz N. (2006). Social paediatrics.. Paediatr Child Health.

